# Geographic variation in the skull morphology of the lesser grison (*Galictis cuja*: Carnivora, Mustelidae) from two Brazilian ecoregions

**DOI:** 10.7717/peerj.9388

**Published:** 2020-11-04

**Authors:** Raissa Prior Migliorini, Rodrigo Fornel, Carlos Benhur Kasper

**Affiliations:** 1 Laboratório de Biologia de Mamíferos e Aves (LABIMAVE), Programa de Pós Graduação em Ciências Biológicas (PPGCB), Universidade Federal do Pampa (UNIPAMPA), São Gabriel, Brazil; 2 Departamento de Ciências Biológicas, Universidade Regional Integrada do Alto Uruguai e das Missões (URI), Erechim, Brazil

**Keywords:** Geometric morphometrics, Intraspecific variation, Sexual dimorphism, Uruguayan savanna, Atlantic Forest

## Abstract

**Background:**

The lesser grison (*Galictis cuja*) is one of the least known carnivores in the Neotropical region. Its wide geographical occurrence and range of habitats could lead to morphological variations along its distribution. So, this study aimed to investigate the variation in skull shape and size of this species, by testing the existence of ecotypes adapted to their respective environments (Uruguayan savanna and Atlantic Forest), as well as its relationship with selected abiotic variables.

**Methods:**

The skulls of 52 museum specimens were photographed in the ventral, dorsal, and lateral views, and were analyzed using geometric morphometric techniques.

**Results:**

We found sexual size dimorphism, with males being larger than females. The shape variation between sexes, as well as between ecoregions, is mostly explained by the effect of allometry. The specimens from Uruguayan savanna are larger than the ones from the Atlantic Forest. Size variation was also significantly correlated to latitude, temperature and precipitation patterns. No correlation between skull shape with geographical distance was detected.

**Discussion:**

Morphometric measurements and diet data of lesser grison in regions from higher latitudes than our sampling show a tendency to heavier individuals, and the consumption of bigger prey compared to Uruguayan savanna. The results indicated the smaller specimens associated to low variability in annual temperature, congruent to Atlantic Forest region. An explanation for observed variation may be related to the “resource rule” but, due the minimal natural history information regards this species, we can just speculate about this.

## Introduction

The lesser grison, *Galictis cuja* (Molina, 1782), is one of the least known mustelids of the Americas, characterized by a thin and elongated body, short legs, and a short, bushy tail (Yensen & Tarifa, 2003; Oliveira, 2009). It is widely distributed across the Neotropical regions, including southern Peru, western Bolivia, central Chile, Paraguay, Uruguay, north and southern Argentina, and east to southeastern Brazil. It inhabits a great variety of habitats from sea level to 4,200 m, such as arid scrub, the Chaco Desert, steppes, wet forest and Andean shrublands (Yensen & Tarifa, 2003). Within Brazil, the lesser grison occurs in almost all ecoregions, including the Cerrado and Caatinga in the northeast, as well as the tropical and subtropical moist broadleaf forest (Atlantic Forest) throughout the eastern seaboard, and the Uruguayan savanna toward the southern region of the country (Bornholdt et al., 2013).

The majority available literature on this species includes information about its trophic ecology in Argentina, Chile, Uruguay, and southern Brazil (Ebensperger, Mella & Simonetti, 1991; Diuk-Wasser & Cassini, 1998; Delibes et al., 2003; Kraus & Rödel, 2004; Zapata et al., 2005; Sade, Rau & Orellana, 2012; Kasper et al., 2015), habitat selection (Zúñiga, Muñoz-Pedreros & Fierro, 2009), and anatomy (Ercoli et al., 2012, 2016). Except for the studies of Zapata et al. (2008) and Schiaffini & Prevosti (2013) and a revision of the morphological and molecular characteristics of the genus *Galictis* (Bornholdt et al., 2013), the cranial morphology of the species has been largely unexplored, even in ecomorphological studies of Mustelidae (Catalano, Ercoli & Prevosti, 2014; Law et al., 2018). Thus, the present study aims to contribute to the knowledge on the intraspecific morphological variation in the skull size and shape of the lesser grison, particularly along its distribution in Brazil.

The cranial, mandibular and dental morphology of the lesser grison are consistent with the general descriptions of weasels (genus *Mustela*) (Zapata et al., 2008). The long and narrow skull features sagittal and nuchal crests that provide extra space for the temporalis muscles to anchor. The jaw is short, and the teeth are specialized for a diet of flesh, to a degree matched only in the cat family (King & Powell, 2007). Relative to other small carnivorans from South America, the lesser grison has a reduced M1, a reduced lingual portion of the upper carnassial P4, a longer and thinner palatal plate that is extended beyond M1, and a longer basicranium (Schiaffini & Prevosti, 2013; Prevosti & Forasiepi, 2018). In addition, the pre- and postglenoid processes are so developed that it is difficult to separate the jaws from the skull, which indicates that this species has a particularly powerful bite (Zapata et al., 2008). This characteristic has been associated with greater efficiency in closing the jaws in mustelids that feed on prey larger than themselves (Christiansen & Wroe, 2007). This is also consistent with some morphofunctional specializations, such as a flexible axial region and strong neck muscles, which give the lesser grison the ability to pursue and hunt its prey through narrow tunnels, as well as to transport relatively heavy prey in the mouth (Yensen & Tarifa, 2003; Ercoli et al., 2016). Its diet consists of small to medium-sized vertebrates, especially lagomorphs, rodents, birds, frogs, lizards, snakes, and their eggs (Yensen & Tarifa, 2003).

The wide geographical distribution of the lesser grison and the range of habitats this mustelid occurs in offer the opportunity to evaluate morphological variations, by testing the existence of ecotypes (Turesson, 1922). Morphometric analyses of several mammalian craniums suggest that patterns of morphological variation in species with large distribution areas might be adaptations to a range of environmental conditions (Gay & Best, 1996), availability of resources (Mcnab, 2010; Schiaffini, 2016a), or to reduce competition with ecologically similar species occurring in sympatry (Bubadué et al., 2015). Thus, we hypothesize that there should be a variation in the size and/or shape of the skull of the lesser grison among populations of different ecoregions throughout the distribution of the species in Brazil, leading to the existence of ecotypes adapted to their respective environments. We also tested the relationships between the skull size variation and selected abiotic variables.

## Materials and Methods

### Sampling and variables

The skulls of museum specimens of the lesser grison were photographed with a Sony DSC-H9 digital camera at a fixed distance of 24 cm, using a support platform. One millimeter graph paper was used as a photographic background for subsequent scale referencing. The photographs were taken and landmarks were digitalized by the same investigator (RPM) to avoid inter-observer error. Only adult skulls with known locality and sufficient integrity to digitalize landmarks that represent the overall skull morphology were included in the statistical analyses. Adult specimens were recognized as those that presented a fully erupted permanent dentition along with a total fusion of the skull sutures (Bornholdt et al., 2013).

**Figure 1:**
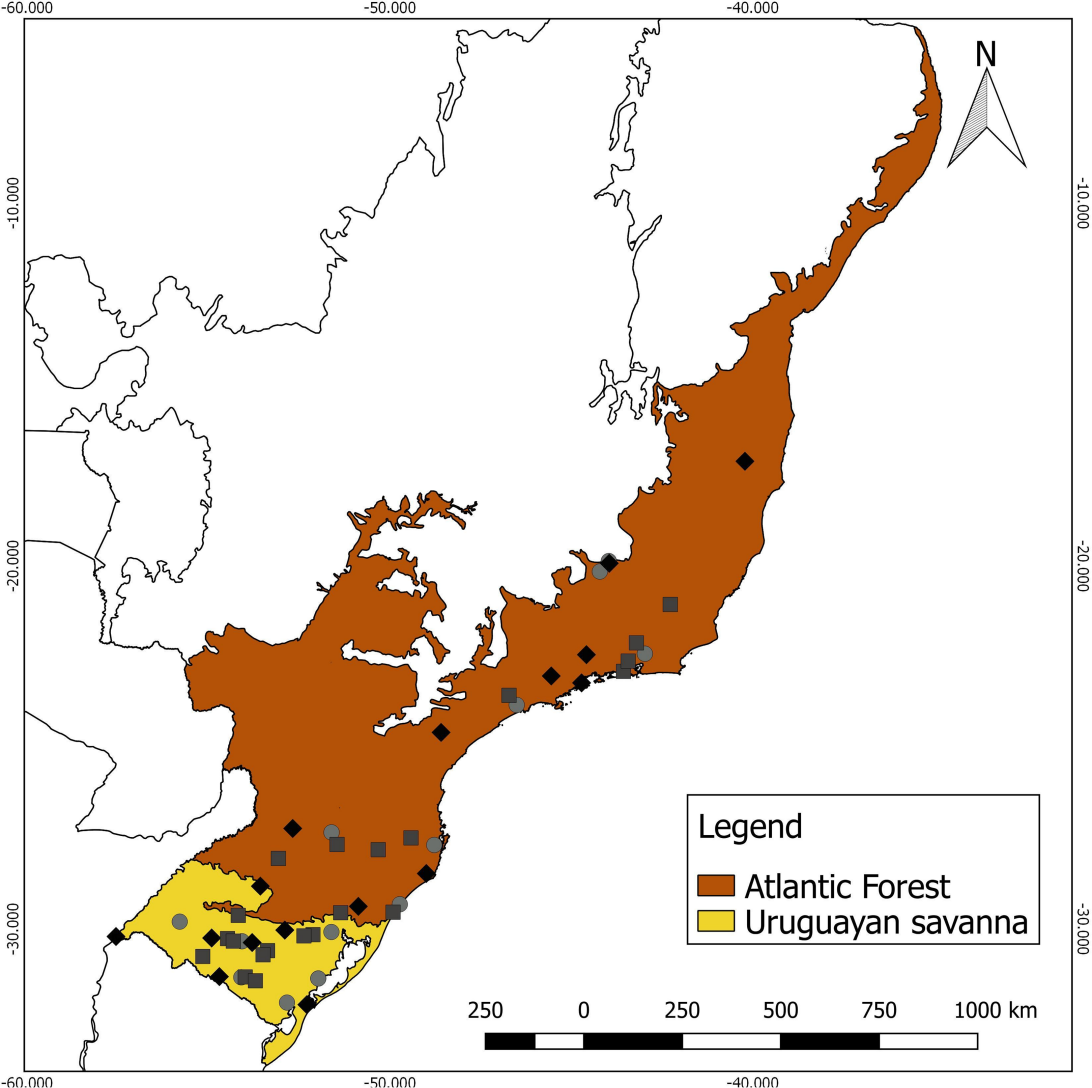
Geographic distribution of the 52 analyzed specimens of *Galictis cuja* (squares, males; diamonds, females; and circles, unsexed), indicating the ecoregions of origin.

Specimens lacking the corresponding geographical coordinates were georeferenced using Google Earth (Google, 2018), using the central coordinate of the municipality cataloged by the collections as the reference. Each specimen was assigned to a Neotropical ecoregion, based on the nomenclature proposed by Olson et al. (2001), using a shapefile in QGIS 2.18.25 (QGIS Development Team, 2018). As the sampling numbers were small, the specimens were grouped into two contrasting major habitat types for analysis (Fig. 1): (a) Uruguayan savanna—where medium/tall grasslands prevail, with sparse shrub and tree formations (11 males, eight females, and eight unsexed); and (b) Atlantic Forest—a forested formation composed of the specimens from the Alto Paraná Atlantic Forest, Araucaria Moist Forest, Serra do Mar Coastal Forest, Southern Atlantic Mangroves, Bahia Interior Forest, and Bahia Coastal Forest ecoregions (10 males, nine females, and six unsexed).

Each specimen was also assigned a value of latitude, as well as 19 bioclimatic variables taken from the WorldClim version 2.1 database (Fick & Hijmans, 2017) at a spatial resolution of 30s (approximately 1 km^2^). This methodological step was realized in QGIS 2.18.25 (QGIS Development Team, 2018).

The examined specimens are housed in the open access mammalian collections of the following institutions: Museu de Zoologia do Pampa (MZPAMPA), located at the Laboratório de Biologia de Mamíferos e Aves, of the Universidade Federal do Pampa, São Gabriel (LABIMAVE-UNIPAMPA); Museu de Ciências Naturais da Fundação Zoobotânica do Rio Grande do Sul, Porto Alegre, Brazil (FZB/RS); Museu de Ciências e Tecnologia da Pontifícia Universidade Católica do Rio Grande do Sul, Porto Alegre, Brazil (MCT-PUCRS); Museu de Ciências Naturais da Universidade Luterana do Brasil, Canoas, Brazil (MCNU-ULBRA); Laboratório de Mamíferos Aquáticos da Universidade Federal de Santa Catarina, Florianópolis, Brazil (LAMAq-UFSC); Museu de Zoologia da Universidade de São Paulo, São Paulo, Brazil (MZUSP); Museu Nacional de História Natural, Rio de Janeiro, Brazil (MNHN-UFRJ); Centro de Coleções Taxonômicas da Universidade Federal de Minas Gerais, Belo Horizonte, Brazil (CCT-UFMG); and Museu Paraense Emílio Goeldi, Belém, Brazil (MPEG). These collections were accessed through contact with the respective curators responsible for them. The list of analyzed specimens is presented in Table S1.

### Geometric morphometric procedures

**Figure 2:**
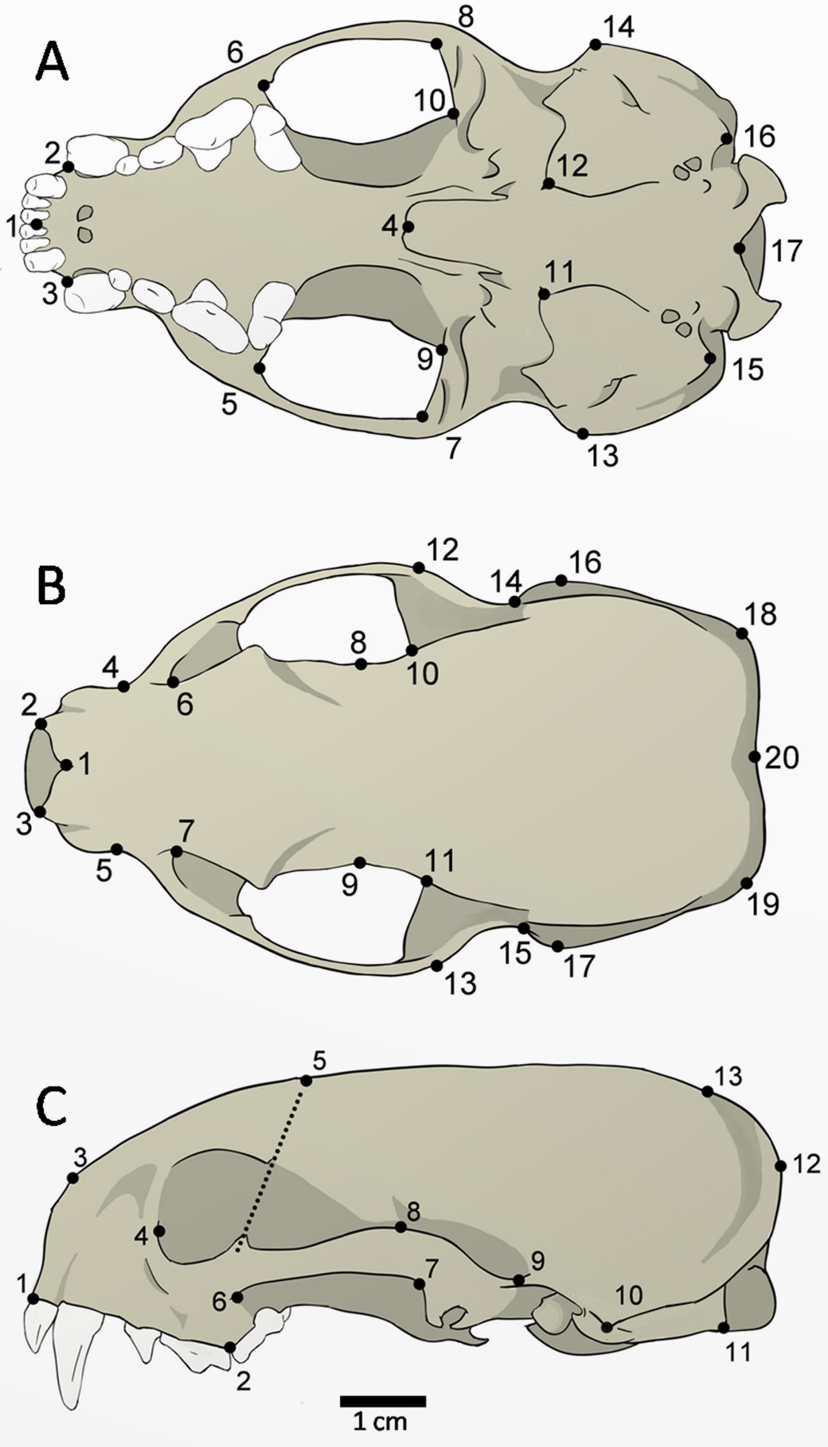
Landmarks digitized in 52 skulls of *Galictis cuja*. Ventral view (A): middle point of the incisive series (1); most anterior point of the canines (2–3); posterior point of the palatine torus (4), temporal-masseter muscle insertion area (5–10); anterior point of the tympanic bulla (11–12); most lateral point of the mastoid process (13–14); paroccipital process (15–16); and ventral point in the foramen magnum (17). Dorsal view (B): anterior point of the nasals in the *sutura internasalis* (1); tips of the nasal process (2–3); point of least width between the maxillae (4–5); the lacrimal foramen (6–7); point of least width between the frontals (8–9); anterior point of the squamous (10–11); most external posterior point of the zygomatic arch (12–13); posterior point of the zygomatic arch (14–15); most lateral point of the mastoid process (16–17); most external points of the lambdoid crest (18–19); and point of the inion (20). Lateral view (C): anterior point of the dentary row (1); point between P4 and M1 (2); most anterior point of the premaxilla and nasal bone suture (3); the lacrimal foramen (4); point of intersection between the dorsal margin of the frontal bone and a line that crosses the ventral and dorsal postorbital processes (5); most lateral point of the jugal on the maxilla (6); most posterior ventral (7) and higher (8) points of the squamosal process; posterior point of the zygomatic arch on the braincase (9); most lateral point of the mastoid process (10); the paroccipital process tip (11); most external point of the lambdoid crest (12); and point of the inion (13).

The skulls of 52 adult specimens (21 males, 17 females, and 14 unsexed) were photographed in the ventral (*n* = 48), dorsal (*n* = 51), and lateral views (*n* = 52) (Fig. 2). The photos were compiled using tpsUtil 1.64 (Rohlf, 2013) and the landmarks were digitalized using tpsDig 2.26 (Rohlf, 2015). The landmarks can be defined as type II (ventral: 1, 2, 3, 4, 5, 6, 7, 8, 9, 10, 11, 12, 15, 16, 17; dorsal: 1, 2, 3, 8, 9, 10, 11, 20; lateral: 1, 2, 3, 10, 11), and type III (ventral: 13, 14; dorsal: 4, 5, 6, 7, 12, 13, 14, 15, 16, 17, 18, 19; lateral: 4, 5, 6, 7, 8, 9, 12, 13), according to Bookstein (1991).

### Data analysis

First, landmark coordinates were superimposed with a generalized Procrustes analysis (GPA) (Dryden & Mardia, 1998), which removes the effects not related to shape, such as position, scale, and orientation. The GPA generates new data from landmark coordinates, the centroid size and shape residuals (Procrustes coordinates). Although extracting the size information from the raw data of landmark coordinates eliminates the variation in size per se, the shape data may still contain a component of size-related shape variation because of the effects of allometry (Klingenberg, 2016). Therefore, the presence of allometry was examined using a multivariate regression of shape on size (Monteiro, 1999). As preliminary analysis, we performed a Shapiro–Wilk test to analyze the normality of size (i.e., centroid size) data (Royston, 1995). So, sexual size dimorphism was evaluated using a Student’s *t*-test since data presented normal distribution for the three views. Sexual differences in shape (i.e., Procrustes coordinates), as well as the interaction between the factors “sex” and “ecoregion”, were tested through a Procrustes analysis of variance (ANOVA) (Adams & Otárola-Castillo, 2013).

To analyze statistical skull size differences between the Uruguayan savanna and Atlantic Forest samples we performed the Student’s *t*-test for the views with normal distribution of measurements and the nonparametric Mann–Whitney test (Hollander & Wolfe, 1973) for the views that did not present normality. The existence of differences in the skull shape between the two distinct ecoregions was tested employing a Procrustes ANOVA (Adams & Otárola-Castillo, 2013). The dimensions of the Procrustes coordinates of shape variation were reduced with a principal component analysis (PCA). The subset of principal components (PCs) sufficient to explain 99% of the total variance was used to perform a linear discriminant analysis (LDA), to compute a leave-one-out cross-validation, which was in turn used to calculate the percentages of correct classifications for both groups (Baylac & Friess, 2005).

The relationship between skull size (i.e., centroid size) and latitude was tested through a multivariate regression (Monteiro, 1999). The association between size and the 19 bioclimatic variables was tested by a single ordinary least-squared (OLS) regression analyses, conducted first separately for each variable (see Table S3). Correlation coefficients (*r*) between the predictor variables were calculated to avoid multicollinearity (Graham, 2003). For those with *r* > 0.7 we applied the stepwise algorithm to keep in the model the variables that provided the best explanation. Thus, a subsequent multiple regression analyses were performed to evaluate the contribution of each predictor variable to size in the presence of the others. The existence of spatial autocorrelation, that is the lack of independence between pairs of observations at given geographical distances (Legendre, 1993), in the size data was evaluated in the residuals of the OLS by the Moran’s *I* index (Diniz-Filho, Bini & Hawkins, 2003).

The distance between the midpoints of each specimen’s locality was used to generate a geographical distance matrix, using the *Geographic Distance Matrix Generator* v 1.2.3 (Ersts, 2018). The residuals of the GPA, originated by the superimposition of the landmark coordinates of the specimens, were used to generate a Mahalanobis distance matrix. The degree of morphological variation was correlated with the geographic distance of the specimens, through an approach similar to the isolation-by-distance model, commonly used in genetic studies (Wright, 1943). For this issue, these matrices were tested by correlation, through an RV coefficient (Heo & Gabriel, 1997).

All statistical analyses and graphs were generated in “R”, version 4.0.0 (R Development Core Team, 2018), using the libraries *MASS* (Venables & Ripley, 2013), *ape* (Paradis, Claude & Strimmer, 2004), *stats* (R Development Core Team, 2018), *ade4* (Dray & Dufour, 2007), *geomorph* (Adams & Otárola-Castillo, 2013), and *letsR* (Vilela & Villalobos, 2015).

## Results

### Sexual dimorphism

The males and females differed in skull size in the ventral (*t* = −5.9755, df = 33.789, *p* < 0.001), dorsal (*t* = −7.006, df = 33.93, *p* < 0.001), and lateral (*t* = −6.853, df = 35.914, *p* < 0.001) views, with males being larger than females. However, the multivariate regression of shape on size revealed that allometry was also significant. The percentages of shapes predicted by size for males and females were 28.2% (*p* = 0.001), 26.7% (*p* = 0.001), and 11.3% (*p* = 0.003) for the ventral, dorsal, and lateral views, respectively. The Procrustes ANOVA confirmed this, showing size as the significant variable explaining sexual dimorphism in the shape for the three views. The effect of the factor “sex” alone was non-significant (ventral: SS < 0.001, MS < 0.001, Rsq = 0.018, *F* = 0.875, *p* = 0.546; dorsal: SS < 0.001, MS < 0.001, Rsq = 0.018, *F* = 0.884, *p* = 0.535; and lateral: SS = 0.002, MS = 0.002, Rsq = 0.028, *F* = 1.123, *p* = 0.285). Based on the absence of interaction between the factors “sex” and “ecoregion” (both in skull size and shape), the males, females, and unsexed specimens were pooled together for the subsequent analysis of variation between ecoregions (see Table S2).

### Skull size and shape between ecoregions

**Figure 3:**
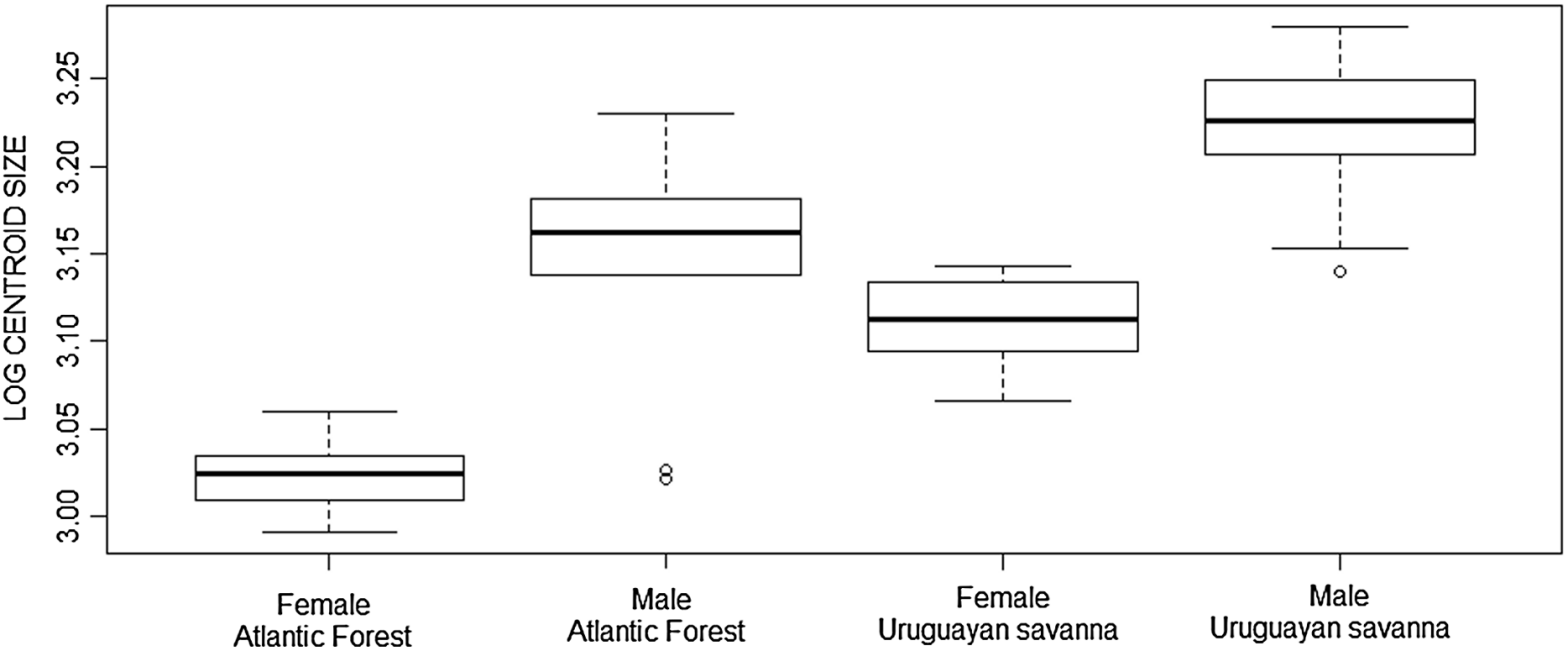
Boxplot of the skull log centroid size of *Galictis cuja* (ventral view) among different ecoregions in Brazil. The black line represents median values and the boxes represent the first interquartile, while the bars represent the second interquartile.

The Student’s *t*-test showed significant differences in skull size between the Atlantic Forest and Uruguayan savanna groups in the ventral view (*t* = −4.413, df = 45.972, *p* < 0.001), as well as the Mann–Whitney test in dorsal (*W* = 0.947, *p* = 0.025), and lateral (*W* = 0.953, *p* = 0.047) views, with the specimens from the Uruguayan savanna being larger than those from the Atlantic Forest, as represented in the boxplot of the ventral view (Fig. 3).

**Figure 4:**
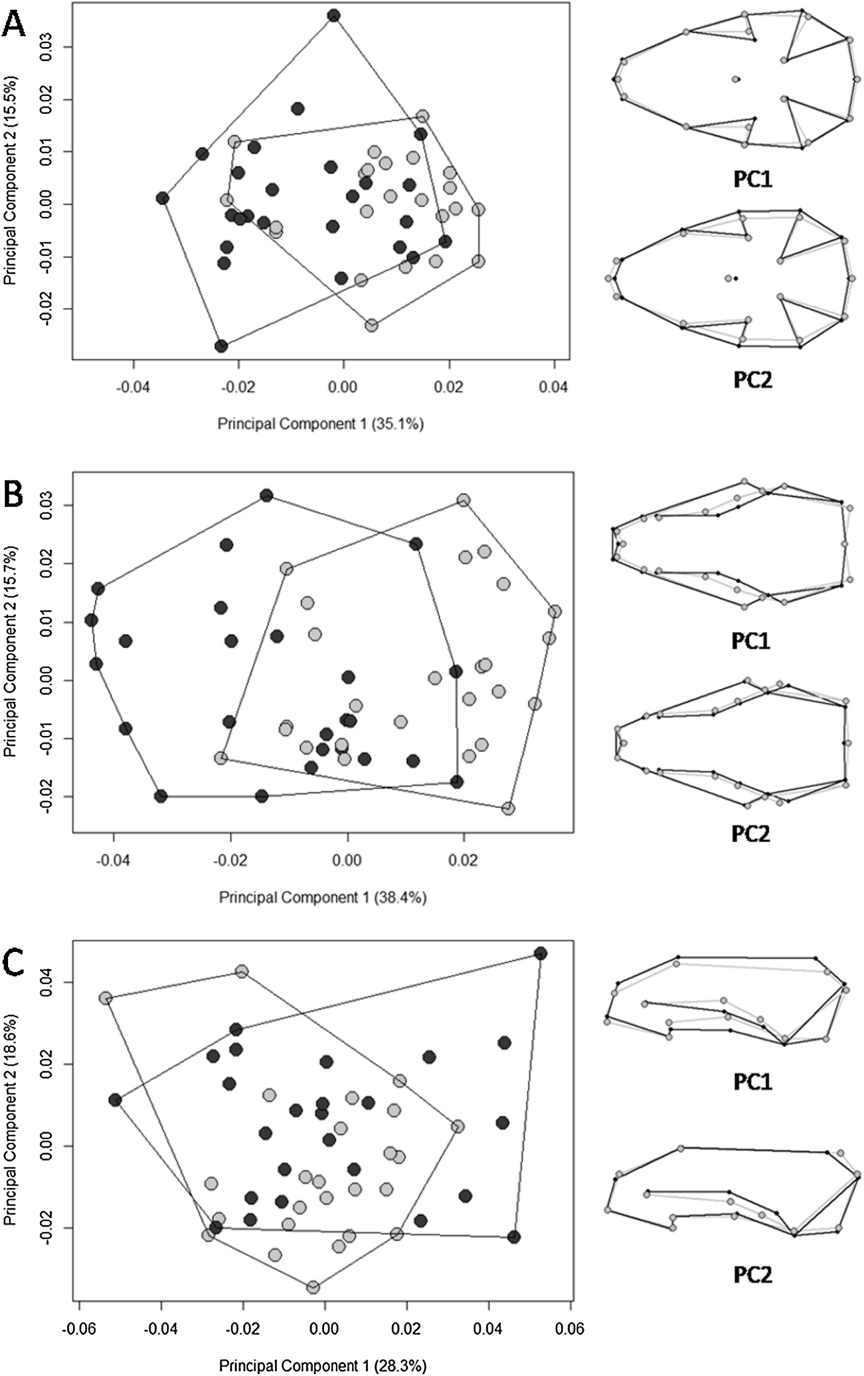
Scatter plots of the two first axes of the principal component analysis (PCA) for *Galictis cuja* specimens from Brazil, based on the Procrustes distances of the consensus shape. Uruguayan savanna individuals are represented in light gray circles and Atlantic Forest in dark gray circles. ****PC1 represents the skull shape variation in the first axis and PC2 represents skull shape variation in the second axis, in the ventral (A), dorsal (B) and lateral (C) views. Positive scores are represented by black lines and negative scores are represented by gray lines.

The multivariate regression of shape on size revealed that allometry was significant for all three views of the skull. The amount of shape predicted by size was 26.4% (*p* = 0.001), 26.6% (*p* = 0.001), and 9.5% (*p* = 0.001) for the ventral, dorsal, and lateral views, respectively. There were significant differences in skull shape; however, according to the Procrustes ANOVA, they are mostly explained by size, except for the dorsal view where the factor “biome” explains 3.6% of the shape variance (SS = 0.002, MS = 0.002, Rsq = 0.036, *F* = 2.563, *p* = 0.006). The first 11 PCs cumulatively explained 95.5% of total variance in the dorsal view; the first 11 explained 95.6% of the variance in the ventral view; and the first 13 explained 95% of variance in the lateral view. Plotting the first PCs with the second, the PCA plot did not cluster groups for any of the skull views (Fig. 4). The percentages of correct classifications for each skull view are shown in Table 1.

**Table 1:** Percentage of correct classification from linear discriminant analysis (LDA) for the skull shape of *Galictis cuja* specimens from two Brazilian ecoregions.

	Uruguayan savanna	Atlantic forest	Overall
Ventral	40	60	56.2
Dorsal	65.3	56	60.7
Lateral	52	48	50

### Abiotic variables

Because of the presence of significant sexual dimorphism in skull size and the allometric component leading to dimorphism in shape, subsequent analyses were conducted with the sexes separated. There was a significant association between size and latitude for both females and males in the three skull views (Table 2).

**Table 2:** Results of Procrustes analysis of variance (ANOVA) test for the relationship between latitude and the skull size of *Galictis cuja* specimens from Brazil.

		SS	MS	Rsq	*F*	*p*
Female	Ventral	8.982	8.982	0.498	13.909	**0.004**
	Dorsal	9.694	9.694	0.465	13.061	**0.003**
	Lateral	8.734	8.734	0.380	9.220	**0.009**
Male	Ventral	21.658	21.658	0.431	13.656	**0.003**
	Dorsal	11.685	11.684	0.188	4.193	0.054
	Lateral	17.579	17.579	0.356	10.547	**0.003**

**Note:**

SS, Sum of squares; MS, mean squares; Rsq, coefficient of determination *R*-squared and *F* value. Significance (*p* < 0.05) is highlighted in bold.

The main results of the OLS are presented in Table 3. Temperature Seasonality explained a significant 57.7% of size variation in ventral and 59.5% in dorsal view of females’ skulls. Temperature Annual Range was the only significant predictor of variance in female lateral view (59.4%) and male dorsal view (38.1%). This last variable, together with Precipitation of Driest Month, Precipitation Seasonality and Precipitation of Wettest Quarter were the most important variables for male ventral view, explaining 75% of size variation. Finally, Precipitation of Wettest Month, Precipitation of Driest Month and Precipitation Seasonality explained 68.2% of size variation for male lateral view.

**Table 3:** Results of single ordinary least-squared (OLS) analyses of skull size of *Galictis cuja* specimens from Brazil and the predictor independent (*r* < 7) bioclimatic variables.

		Bioclimatic variable	*p*
Female	Ventral	Temperature Seasonality	**<0.001**
	Dorsal	Temperature Seasonality	**0.001**
		Max Temperature of Warmest Month	0.119
	Lateral	Max Temperature of Warmest Month	0.158
		Temperature Annual Range	**0.001**
Male	Ventral	Temperature Annual Range	**0.022**
		Mean Temperature of Wettest Quarter	0.455
		Mean Temperature of Driest Quarter	0.831
		Mean Temperature of Coldest Quarter	0.492
		Precipitation of Driest Month	**0.027**
		Precipitation Seasonality	**0.025**
		Precipitation of Wettest Quarter	**0.004**
	Dorsal	Temperature Annual Range	**0.002**
	Lateral	Mean Temperature of Wettest Quarter	0.960
		Precipitation of Wettest Month	**<0.001**
		Precipitation of Driest Month	**<0.001**
		Precipitation Seasonality	**<0.001**

**Note:**

Significance (*p* < 0.05) is highlighted in bold.

No significant (*p* > 0.05) spatial autocorrelation in the OLS residuals were found (see Table S4). The results also showed no significant correlations between the geographical distance and Procrustes distances of the consensus shape for both males (ventral: *p* = 0.271; dorsal: *p* = 0.417; lateral: *p* = 0.176) and females (ventral: *p* = 0.382; dorsal: *p* = 0.571; lateral: *p* = 0.198).

## Discussion

### Sexual dimorphism

Our results indicate sexual dimorphism in the skull size of the lesser grison, with males being larger than females. This is not surprising, as sexual size dimorphism within the Mustelidae family has been reported, especially among smaller species (Noonan et al., 2016; Law & Mehta, 2018). Small mustelids, such as the lesser grison, are predominately obligate carnivores, feeding almost exclusively on vertebrates (Zapata et al., 2005). In this species the dispersion of food resources would promote intrasexual selection for territories, resulting in mating systems where males must compete for access to females, which confers a selective advantage to larger males (Noonan et al., 2016). Nevertheless, increased cranial size dimorphism could reduce dietary competition between the sexes, rendering niche divergence a mechanism in the maintenance of the evolution of sexual dimorphism in extant mustelids (Law & Mehta, 2018).

Allometry is a pervasive aspect of morphological variation in mammals, in which large differences in size are typically accompanied by differences in shape because of the covariation in these traits (Klingenberg, 2016). This association became evident for the lesser grison, as the shape variation between sexes we found was mainly related to their differences in size. The most obvious changes are seen in the more developed lambdoid crest and mastoid process in males (Fig. S1). This is consistent with the effect of allometry on the muscular insertion areas described for carnivores in general, and weasels in particular, where the smaller females usually differ from males by presenting reduced muscular insertion processes (Ercoli, 2017).

### Skull size and shape between ecoregions

Our hypothesis on the existence of ecotypes according to different ecoregions was supported; we found significantly larger skull sizes in specimens from the Uruguayan savanna than those in the Atlantic Forest. Overall, the PCA displayed patterns of shape overlap between specimens from both ecoregions in all three views (Fig. 4). A relative differentiation was only seen along the first axis for the dorsal view, which placed the specimens from the Atlantic Forest mostly in the negative scores, representing a skull with the region that encompasses the points of least width between frontals, the anterior points of the squamous, to the posterior point of the zygomatic arch, wider than that of specimens from the Uruguayan savanna. All of the observed skull shape variations were significantly explained by allometry. Both Procrustes ANOVA and the percentage of correct classification indicated that the differences between Atlantic Forest and Uruguayan savanna in the skull shape are small. The percentage of correct classification for skull shape showed values near 50%, the expected by chance in two possibilities, reinforcing the similarity between the ecoregions.

Despite the particular compositions of the ecoregions clustered as Atlantic Forest (Olson et al., 2001), because of our small sample size and since those ecoregions are all associated to forested formations (Instituto Brasileiro de Geografia Estatística (IBGE), 1993), we decided to focus the analysis on the comparison of two major contrasting habitats (i.e., moist broadleaf forests *versus* grasslands, savannas and shrublands). Intraspecific variation has already been observed in some characteristics of the lesser grison, such as fur color, with the yellowish specimens found mainly in the open and drier landscapes, for example, savannahs and grasslands, which is assumed to be an adaptation favored as camouflage (Bornholdt et al., 2013). Patterns of morphological change associated with contrasting habitats, in which different selective pressures leads to changes in skull shape and size, have also been observed in other mustelids (Hernández-Romero, Guerrero & Valdespino, 2015). The lesser grison is usually described as a generalist species regarding habitat requirements (Yensen & Tarifa, 2003; Schiaffini, 2016b), and it appears to be unusual in most habitats (Kasper et al., 2013). However, more data on the species ecology and increased samplings (especially from its northern distribution) are needed to better understand its skull size variation.

When two morphologically and or/ecologically similar species geographically overlap, a change in size or morphology is expected to minimize competition (Brown & Wilson, 1956). Competition is assumed to occur mostly among closely related species, but may also take place among species from different clades with similar ecologies (Van Valkenburgh, 1999). Throughout the ecoregions we sampled, there are no other similar small mustelids that could compete with the lesser grison for the same resources, with the only other hypercarnivorous species in sympatry being the felids (Reis et al., 2011). However, despite its food indices having up to 95% similarity with small cats, as reported in southern Brazil (Kasper et al., 2015), these species use different foraging strategies. Cats generally lie in ambush by trails or burrow entrances waiting for the prey to appear (Kitchener, Van Valkenburgh & Yamaguchi, 2010). Thus, it can be argued that character displacement does not provide the most comprehensive explanation for the lesser grison’s skull size differentiation between the different ecoregions.

The body size of small weasel-like mammals is probably most strongly influenced by the most workable balance between hunting efficiency and energy balance under local conditions (King & Powell, 2007). In other species of Mustelidae, such as an assemblage composed of three New World sympatric least weasels, it is assumed that the force driving the morphological convergence in their canine size may simply be a local adaptation for hunting similar resources (Meiri, Simberloff & Dayan, 2011). Despite its wide distribution, the lesser grison’s food habits are poorly understood in most of its habitat range, precluding a comparison of patterns of prey consumption from the Uruguayan savanna and Atlantic Forest groups.

### Abiotic variables

We also found a significant association between skull size and latitude, following the predictions of Bergmann’s rule. This indicates that within warm-blooded (endothermic) species, individuals from colder climates or higher latitudes are generally larger than those from warmer regions or lower latitudes (Bergmann, 1847). Data on morphometric measurements from the lesser grison’s distribution further south of our sampling, that is higher latitudes, comes from Buenos Aires Province (Vidal et al., 2016). The authors recorded body mass outside the range reported in the literature, with specimens weighing up to 2.8 kg. The average male body mass of 2.12 kg is greater than the mean weight of the specimens available in the collection database we sampled from the Uruguayan savanna (1.74 kg), which was also greater than that from the Atlantic Forest (1.41 kg). These values may be indicative of a trend in the skull size following this pattern throughout its distribution.

Although Bergmann’s rule has been extensively discussed, there is no general agreement about its validity. The heat retention explanation and mass-specific rates have been criticized for their inconsistent ecological realities (McNab, 1999). Therefore, another proposal for trends in size was made by Mcnab (2010), who called it the “resource rule”, according to which the spatial variation in mammal body size could be explained by the availability and characteristics of the consumed resources. Mustelids, such as the lesser grison, have higher energy needs than expected for their body mass, which may require a larger prey size to satisfy their energetic requirements (Brown & Lasiewski, 1972). In Argentina, the lesser grison’s diet consists mostly of introduced lagomorphs, which seems consistent with its ability to subdue prey larger than expected for its body size, even in burrows and nests (Ebensperger, Mella & Simonetti, 1991; Delibes et al., 2003). A study from southern Brazil (Kasper et al., 2015) suggested a diet based on smaller rodents, and despite the high abundance of *Lepus europaeus* (Pallas, 1778) in the region (Kasper et al., 2012), there is no evidence of its consumption. Unfortunately, the lack of information regarding the diet of the lesser grison from its Brazilian northern distribution, comprising a single mention based on one fecal sample (Rocha-Mendes et al., 2010), allows us to speculate that those specimens became smaller because they feed on smaller prey.

Skull size variation of lesser grison also seems to be related to temperature and precipitation patterns. Temperature Annual Range and Seasonality showed a positive correlation with skull size, indicating larger specimens occurring in regions with greater variability in annual temperature. The ecoregion analyses showed similar results, once the Atlantic Forest (i.e., moist broadleaf forest) is characterized by low variability in annual temperature and contains the smaller specimens. An environmental niche modeling indicated higher predictive values for lesser grison distribution along regions with highly variable precipitation and temperature (Schiaffini, 2016b). It was proposed that the species presence in cold zones might be related to the consumption of large number of preys to maintain a constant body temperature. As the lesser grison seems to be larger in the southern colder ecoregions, it is possible to suppose that it can prey upon a wider range of prey sizes (Ebensperger, Mella & Simonetti, 1991; Delibes et al., 2003) as a way to overcome the species poor thermal balance.

Our results revealed no significant correlation between geographical and morphological distance. This indicates that the skull shape variation did not follow a pattern similar to the isolation-by-distance model, which predicts an exponential decrease in the genetic similarity between populations, as the geographic distance between them increases (Wright, 1943). Morphological variation in some Neotropical mustelids is explained by the presence of different evolutionary units delimited by geographical barriers (Hernández-Romero, Guerrero & Valdespino, 2015). However, the lesser grison seems to occur continuously and uniformly along the sampled regions, with no apparent geographical barriers. Our result is also congruent with the current research on genetic variability which does not suggest the recognition of subspecies (Bornholdt et al., 2013).

## Conclusions

This study adds new information on the skull size and shape variation of the lesser grison in Brazil. Sexual size dimorphism was observed, with males being larger than females; this is an expected pattern for a small mustelid. The shape variation between the sexes, as well as between ecoregions, is mostly explained by the effect of allometry, and no correlation with geographical distance was detected.

The specimens from the Uruguayan savanna were significantly larger than those from the Atlantic Forest, and the size variation was also significantly correlated to latitude, as well as temperature and precipitation patterns. The morphometric measurement data on regions from higher latitudes than our sampling showed a tendency toward heavier individuals, as well as the consumption of bigger prey, compared to those from the Uruguayan savanna. Unfortunately, its minimal natural history information (Poo-Muñoz et al., 2014) only allows us to speculate that the lesser grison’s skull size variation pattern follows the “resource rule”.

Although relatively common in most parts of Brazil, some ecoregions are not well represented in scientific Brazilian collections as they are hampered by the predominance of young specimens and individuals without cataloged origins. In addition, to completely understand the variations in the skull size and shape of the lesser grison, future studies should focus on gathering information on the species ecological aspects, especially from its northern distribution.

## Supplemental Information

10.7717/peerj.9388/supp-1 Supplemental Information 1 Analyzed specimens of *Galictis cuja*, with their corresponding collection identification, sex (female (F); male (M); unknown (?)), locality of origin and geographical coordinates. They are housed at the following Brazilian institutions: Museu de Zoologia do Pampa (MZPAMPA); Museu de Ciências Naturais da Fundação Zoobotânica do Rio Grande do Sul (FZB/RS); Museu de Ciências e Tecnologia da Pontifícia Universidade Católica do Rio Grande do Sul (MCT-PUCRS); Museu de Ciências Naturais da Universidade Luterana do Brasil (MCNU); Laboratório de Mamíferos Aquáticos da Universidade Federal de Santa Catarina (LAMAq-UFSC); Museu de Zoologia da Universidade de São Paulo (MZUSP); Museu Nacional de História Natural (MNHN); Centro de Coleções Taxonômicas da Universidade Federal de Minas Gerais (CCT-UFMG) and Museu Paraense Emílio Goeldi (MPEG).

10.7717/peerj.9388/supp-2 Supplemental Information 2 The results of Procrustes ANOVA test for the interaction between sex and ecoregion on skull shape and size of 52 *Galictis cuja* specimens from Brazil. Degrees of freedom (Df), sum of squares (SS), mean squares (MS), coefficient of determination R-squared (Rsq), F value and significance (*p*).

10.7717/peerj.9388/supp-3 Supplemental Information 3 Results of single ordinary least-squared (OLS) regression analyses of skull size of 52 *Galictis cuja* specimens and bioclimatic variables separately. Significance (*p* < 0.05) is highlighted in bold.

10.7717/peerj.9388/supp-4 Supplemental Information 4 Results of spatial autocorrelation in the OLS residuals of *Galictis cuja* specimens in ventral (A = male, A’ = female), dorsal (B = male, B’ = female) and lateral (C = male, C’ = female) skull views. Significance (*p* < 0.05) is highlighted in bold.

10.7717/peerj.9388/supp-5 Supplemental Information 5 Scatter plots of the two first axes of the principal component analysis (PCA) for *Galictis cuja* males (light gray circles) and females (dark gray circles) in Brazil, based on the Procrustes distances of the consensus shape. PC1 represents the skull shape variation in the first axis and PC2 represents skull shape variation in the second axis, in the ventral (A), dorsal (B) and lateral (C) views. Positive scores are represented by black lines and negative scores are represented by gray lines.

## Additional Information and Declarations

### Data Availability

The following information was supplied regarding data availability: The raw measurements, specimen and deposition information are available in the Supplemental Files.
