# Supplementary material for: Geographic variation in the skull morphology of the lesser grison (Galictis cuja: Carnivora, Mustelidae) from two Brazilian ecoregions
Source: PeerJ. 2020 Nov 4;8:e9388. doi: 10.7717/peerj.9388 (PMC7648447; doi:10.7717/peerj.9388)
Supplement: Supplemental Information 1 — They are housed at the following Brazilian institutions: Museu de Zoologia do Pampa (MZPAMPA); Museu de Ciências Naturais da Fundação Zoobotânica do Rio Grande do Sul (FZB/RS); Museu de Ciências e Tecnologia da Pontifícia Universidade Católica do Rio Grande do Sul (MCT-PUCRS); Museu de Ciências Naturais da Universidade Luterana do Brasil (MCNU); Laboratório de Mamíferos Aquáticos da Universidade Federal de Santa Catarina (LAMAq-UFSC); Museu de Zoologia da Universidade de São Paulo (MZUSP); Museu Nacional de História Natural (MNHN); Centro de Coleções Taxonômicas da Universidade Federal de Minas Gerais (CCT-UFMG) and Museu Paraense Emílio Goeldi (MPEG). [file peerj-08-9388-s001.docx]

**Table S1.** **Analyzed specimens of *Galictis cuja*, with their corresponding collection identification, sex (female (F); male (M); unknown (?)), locality of origin and geographical coordinates.** They are housed at the following Brazilian institutions: Museu de Zoologia do Pampa (MZPAMPA); Museu de Ciências Naturais da Fundação Zoobotânica do Rio Grande do Sul (FZB/RS); Museu de Ciências e Tecnologia da Pontifícia Universidade Católica do Rio Grande do Sul (MCT-PUCRS); Museu de Ciências Naturais da Universidade Luterana do Brasil (MCNU); Laboratório de Mamíferos Aquáticos da Universidade Federal de Santa Catarina (LAMAq-UFSC); Museu de Zoologia da Universidade de São Paulo (MZUSP); Museu Nacional de História Natural (MNHN); Centro de Coleções Taxonômicas da Universidade Federal de Minas Gerais (CCT-UFMG) and Museu Paraense Emílio Goeldi (MPEG).

| Collection | Specimen ID | Sex | Locality of origin | Geographical coordinates | Ecoregion* |
| --- | --- | --- | --- | --- | --- |
| MZPAMPA | M0004 | F | Cachoeira do Sul, RS | 30°1’60”S / 52°53’60”W | Alto Paraná Atlantic Forest |
|  | M0005 | ? | Santa Margarida do Sul, RS | 30°20’24”S / 54°04’48”W | Uruguayan savanna |
|  | M0102 | M | Rosário do Sul, RS | 30°45’30”S / 55°09’42”W | Uruguayan savanna |
|  | M0116 | ? | São Gabriel, RS | 30°15’58”S / 54°28’46”W | Uruguayan savanna |
|  | M0387 | F | Vila Nova do Sul, RS | 30°22’32”S / 53°48’0.5”W | Uruguayan savanna |
|  | M0452 | M | Minas do Leão, RS | 30°09’27”S / 52°07’27”W | Uruguayan savanna |
|  | M0531 | M | São Gabriel, RS | 30°20’09”S / 54°19’12”W | Uruguayan savanna |
|  | M0542 | F | Rosário do Sul, RS | 30°15’S / 54°55’W | Uruguayan savanna |
|  | M0604 | M | Pantano Grande, RS | 30°11’27”S / 52°22’26”W | Uruguayan savanna |
|  | M0611 | ? | Eldorado do Sul, RS | 30°05’02”S / 51°36’58”W | Uruguayan savanna |
|  | M0618 | ? | Pedro Osório, RS | 32°01’42”S / 52°50’27”W | Uruguayan savanna |
|  | M0688 | M | Caçapava do Sul, RS | 30°42’43”S / 53°29’58”W | Uruguayan savanna |
|  | M0691 | M | Caçapava do Sul, RS | 30°35’50”S / 53°22’35”W | Uruguayan savanna |
|  | M0692 | M | Bagé, RS | 31°19’24”S / 53°59’27”W | Uruguayan savanna |
|  | M0694 | M | Candiota, RS | 31°25’40”S / 53°42’39”W | Uruguayan savanna |
| FZB/RS | MCN2512 | ? | Passo de Torres, SC | 29°19’15”S / 49°43’50”W | Uruguayan savanna |
|  | MCN2765 | F | Bagé, RS | 31°18’50”S / 54°0.4’74”W | Uruguayan savanna |
|  | MCN3065 | M | RS 389, Km 56, RS | 29°32’19”S / 49°55’2.3”W | Uruguayan savanna |
| MCT-PUCRS | MCP1787 | M | RS 446, Bom Princípio, RS | 29°33’11.7”S / 51°21’42”W | Alto Paraná Atlantic Forest |
|  | MCP1820 | ? | BR-290, Alegrete, RS | 29°47’29.5”S / 55°47’48.2”W | Uruguayan savanna |
| MCNU | MCNU071 | F | Barra do Quaraí, RS | 30°12’32.4”S / 57°33’11”W | Uruguayan savanna |
|  | MCNU627 | M | São Pedro do Sul, RS | 29°37’36”S / 54°10’59”W | Uruguayan savanna |
|  | MCNU757 | ? | Bagé, RS | 31°19’60”S / 54°6’0”W | Uruguayan savanna |
|  | MCNU1829 | M | Chapada, RS | 28°3’27.6”S / 53°4’6.3”W | Uruguayan savanna |
|  | MCNU2775 | ? | Rio do Peixe, Ouro, SC | 27°20’23”S / 51°36’45.6”W | Araucaria Moist Forest |
|  | MCNU3720 | F | BR 287, Km 22, RS | 28°49’18”S / 53°34’29”W | Uruguayan savanna |
|  | MCNU3722 | F | Rio Grande, RS | 32°05’10”S / 52°17’35”W | Uruguayan savanna |
| LAMAq-UFSC | UFSC746 | ? | Santo Amaro da Imperatriz, SC | 27°41’8.4”S / 48°46’54”W | Serra do Mar Coastal Forest |
|  | UFSC785 | F | Tubarão, RS | 28°28’19”S / 49°0’53”W | Serra do Mar Coastal Forest |
|  | UFSC870 | M | Rio dos Bugres, SC | 27°29’31”S / 49°25’20.7”W | Serra do Mar Coastal Forest |
|  | UFSC4830 | F | SCT 480, trilha do Pitoco, SC | 27°13’54.8”S / 52°40’57”W | Araucaria Moist Forest |
|  | UFSC5706 | M | Lages, SC | 27°48’56.5”S / 50°19’35”W | Araucaria Moist Forest |
| MZUSP | 000230 | ? | São Lourenço do Sul, RS | 31°21’55”S / 51°58’42”W | Uruguayan savanna |
|  | 000978 | F | Ipiranga, SP | 24°35’08”S / 48°35’35”W | Serra do Mar Coastal Forest |
|  | 001247 | M | Três Rios, RJ | 22°07’00”S / 43°12’33”W | Serra do Mar Coastal Forest |
|  | 006463 | M | Butantã, SP | 23°33’48.5”S / 46°43’15.6”W | Serra do Mar Coastal Forest |
|  | 009633 | ? | Santos, SP | 23°49’41.5”S / 46°30’30.3”W | Serra do Mar Coastal Forest |
|  | 001004 | ? | São Lourenço do Sul, RS | 31°21’55”S / 51°58’42”W | Uruguayan savanna |
| MNHN | 1882 | M | Campo Grande, RJ | 22°54’12.9”S / 43°33’58.4”W | Serra do Mar Coastal Forest |
|  | 3127 | F | Itatiaia, RJ | 22°26’34.9”S / 44°35’14.5”W | Alto Paraná Atlantic Forest |
|  | 3129 | ? | Teresópolis, RJ | 22°25’1.0”S / 42°58’32.2”W | Serra do Mar Coastal Forest |
|  | 8236 | F | Pedra Branca, Paraty, RJ | 23°13’12.2”S / 44°43’13.7”W | Southern Atlantic Mangroves |
|  | 29983 | F | Fazenda Serra Azul, BA | 17°6’50.4”S / 40°13’4.6”W | Bahia Coastal Forest |
|  | 29984 | F | Taubaté, SP | 23°1’13.4”S / 45°33’22.7”W | Serra do Mar Coastal Forest |
|  | 29985 | M | Tinguá, Nova Iguaçu, RJ | 22°36’12.6”S / 43°26’8.4”W | Serra do Mar Coastal Forest |
|  | 30001 | M | São João da Glória, MG | 21°3’3.3”S / 42°15’26.9”W | Bahia Interior Forest |
| CCT-UFMG | UFMG3824 | F | Belo Horizonte, MG | 19°55’28.1”S / 43°56’6.8”W | Bahia Interior Forest |
|  | UFMG3935 | ? | Sítio Braúna, Pampulha, MG | 19°51’44”S / 43°58’14”W | Bahia Interior Forest |
|  | UFMG3978 | ? | Brumadinho, MG | 20°9’4.2”S / 44°12’2.6”W | Bahia Interior Forest |
| MPEG | 22188 | M | Barracão, RS | 27°40’18”S / 51°27’38”W | Araucaria Moist Forest |
|  | 22229 | F | Cachoeira do Sul, RS | 30°1’60”S / 52°53’60”W | Alto Paraná Atlantic Forest |
|  | 22230 | F | Gramado, RS | 29°22’43”S / 50°52’26”W | Araucaria Moist Forest |

*According the nomenclature proposed by Olson et al. (2001).
