# Supplementary material for: Geographic variation in the skull morphology of the lesser grison (Galictis cuja: Carnivora, Mustelidae) from two Brazilian ecoregions
Source: PeerJ. 2020 Nov 4;8:e9388. doi: 10.7717/peerj.9388 (PMC7648447; doi:10.7717/peerj.9388)
Supplement: Supplemental Information 2 — Degrees of freedom (Df), sum of squares (SS), mean squares (MS), coefficient of determination R-squared (Rsq), F value and significance (p). [file peerj-08-9388-s002.docx]

**Table S2. The results of Procrustes ANOVA test for the interaction between sex and ecoregion on skull shape and size of 52 *Galictis cuja* specimens from Brazil.** Degrees of freedom (Df), sum of squares (SS), mean squares (MS), coefficient of determination R-squared (Rsq), F value and significance (*p*).

|  |  | Df | SS | MS | Rsq | F | *p* |
| --- | --- | --- | --- | --- | --- | --- | --- |
| Shape | **Ventral** | 1 | <0.001 | <0.001 | 0.020 | 0.968 | 0.354 |
|  | **Dorsal** | 1 | <0.001 | <0.001 | 0.012 | 0.617 | 0.664 |
|  | **Lateral** | 1 | <0.001 | <0.001 | 0.010 | 0.394 | 0.874 |
| Size | **Ventral** | 1 | 0.001 | 0.001 | <0.001 | <0.001 | 0.979 |
|  | **Dorsal** | 1 | 0.135 | 0.135 | <0.001 | 0.078 | 0.797 |
|  | **Lateral** | 1 | 0.297 | 0.297 | 0.001 | 0.223 | 0.641 |
