# Supplementary material for: Geographic variation in the skull morphology of the lesser grison (Galictis cuja: Carnivora, Mustelidae) from two Brazilian ecoregions
Source: PeerJ. 2020 Nov 4;8:e9388. doi: 10.7717/peerj.9388 (PMC7648447; doi:10.7717/peerj.9388)
Supplement: Supplemental Information 3 — Significance (p < 0.05) is highlighted in bold. [file peerj-08-9388-s003.docx]

**Table S3. Results of single ordinary least-squared (OLS) regression analyses of skull size of 52 *Galictis cuja* specimens and bioclimatic variables separately.** Significance (*p* < 0.05) is highlighted in bold.

| Bioclimatic variable | Female | | | Male | | |
| --- | --- | --- | --- | --- | --- | --- |
|  | **Ventral** | **Dorsal** | **Lateral** | **Ventral** | **Dorsal** | **Lateral** |
| BIO1 | Rsq = - 0.041  *p* = 0.538 | Rsq = -0.041  *p* = 0.555 | Rsq = -0.037  *p* = 0.523 | Rsq = -0.006  *p* = 0.359 | Rsq = -0.052  *p* = 0.823 | Rsq = 0.034  *p* = 0.206 |
| BIO2 | Rsq = -0.057  *p* = 0.675 | Rsq = -0.049  *p* = 0.624 | Rsq = -0.064  *p* = 0.857 | Rsq = -0.0007  *p* = 0.334 | Rsq = 0.042  *p* = 0.192 | Rsq = -0.020  *p* = 0.446 |
| BIO3 | Rsq = 0.463  ***p* = 0.002** | Rsq = 0.448  ***p* = 0.001** | Rsq = 0.348  ***p* = 0.007** | Rsq = 0.252  ***p* = 0.014** | Rsq = 0.056  *p* = 0.161 | Rsq = 0.213  ***p* = 0.020** |
| BIO4 | Rsq = 0.577  ***p* < 0.001** | Rsq = 0.548  ***p* <0.001** | Rsq = 0.512  ***p* < 0.001** | Rsq = 0.580  ***p* < 0.001** | Rsq = 0.275  ***p* = 0.010** | Rsq = 0.480  ***p* < 0.001** |
| BIO5 | Rsq = 0.155  *p* = 0.073 | Rsq = 0.183  ***p* = 0.048** | Rsq = 0.197  ***p* = 0.042** | Rsq = 0.068  *p* = 0.139 | Rsq = 0.111  *p* = 0.082 | Rsq = -0.007  *p* = 0.366 |
| BIO6 | Rsq = 0.027  *p* = 0.252 | Rsq = 0.012  *p* = 0.291 | Rsq = 0.059  *p* = 0.176 | Rsq = 0.129  *p* = 0.066 | Rsq = 0.013  *p* = 0.277 | Rsq = 0.170  ***p* = 0.035** |
| BIO7 | Rsq = 0.446  ***p* = 0.002** | Rsq = 0.429  ***p* = 0.002** | Rsq = 0.561  ***p* < 0.001** | Rsq = 0.562  ***p* < 0.001** | Rsq = 0.381  ***p* = 0.002** | Rsq = 0.439  ***p* < 0.001** |
| BIO8 | Rsq = 0.004  *p* = 0.319 | Rsq = 0.023  *p* = 0.258 | Rsq = -0.024  *p* = 0.447 | Rsq = 0.208  ***p* = 0.024** | Rsq = 0.091  *p* = 0.105 | Rsq = 0.153  ***p* = 0.044** |
| BIO9 | Rsq = 0.048  *p* = 0.205 | Rsq = -0.030  *p* = 0.477 | Rsq = -0.066  *p* = 0.941 | Rsq = 0.166  ***p* = 0.042** | Rsq = 0.071  *p* = 0.133 | Rsq = 0.048  *p* = 0.172 |
| BIO10 | Rsq = 0.054  *p* = 0.193 | Rsq = 0.068  *p* = 0.160 | Rsq = 0.044  *p* = 0.205 | Rsq = -0.019  *p* = 0.435 | Rsq = 0.006  *p* = 0.302 | Rsq = -0.048  *p* = 0.794 |
| BIO11 | Rsq = 0.134  *p* = 0.089 | Rsq = 0.124  *p* = 0.090 | Rsq = 0.128  *p* = 0.086 | Rsq = 0.168  ***p* = 0.040** | Rsq = 0.014  *p* = 0.273 | Rsq = 0.206  ***p* = 0.022** |
| BIO12 | Rsq = -0.065  *p* = 0.79 | Rsq = -0.066  *p* = 0.987 | Rsq = -0.066  *p* = 0.998 | Rsq = -0.041  *p* = 0.633 | Rsq = - 0.009  *p* = 0.378 | Rsq = -0.028  *p* = 0.514 |
| BIO13 | Rsq = 0.191  ***p* = 0.050** | Rsq = 0.140  *p* = 0.076 | Rsq = 0.082  *p* = 0.139 | Rsq = 0.456  ***p* < 0.001** | Rsq = 0.263  ***p* = 0.012** | Rsq = 0.378  ***p* = 0.001** |
| BIO14 | Rsq = 0.095  *p* = 0.13 | Rsq = 0.127  *p* = 0.087 | Rsq = 0.061  *p* = 0.173 | Rsq = 0.388  ***p* = 0.001** | Rsq = 0.134  *p* = 0.062 | Rsq = 0.305  ***p* = 0.005** |
| BIO15 | Rsq = 0.209  ***p* = 0.042** | Rsq = 0.205  ***p* = 0.038** | Rsq = 0.106  *p* = 0.109 | Rsq = 0.382  ***p* = 0.002** | Rsq = 0.146  *p* = 0.053 | Rsq = 0.297  ***p* = 0.006** |
| BIO16 | Rsq = 0.167  *p* = 0.064 | Rsq = 0.133  *p* = 0.082 | Rsq = 0.066  *p* = 0.164 | Rsq = 0.472  ***p* < 0.001** | Rsq = 0.268  ***p* = 0.011** | Rsq = 0.392  ***p* = 0.001** |
| BIO17 | Rsq = 0.050  *p* = 0.202 | Rsq = 0.096  *p* = 0.12 | Rsq = 0.029  *p* = 0.243 | Rsq = 0.358  ***p* = 0.003** | Rsq = 0.118  *p* = 0.076 | Rsq = 0.281  ***p* = 0.007** |
| BIO18 | Rsq = 0.105  *p* = 0.118 | Rsq = 0.076  *p* = 0.148 | Rsq = 0.017  *p* = 0.273 | Rsq = 0.76  ***p* < 0.001** | Rsq = 0.341  ***p* = 0.004** | Rsq = 0.476  ***p* < 0.001** |
| BIO19 | Rsq = 0.075  *p* = 0.157 | Rsq = 0.109  *p* = 0.106 | Rsq = 0.017  *p* = 0.274 | Rsq = 0.347  ***p* = 0.003** | Rsq = 0.113  *p* = 0.080 | Rsq = 0.269  ***p* = 0.009** |

BIO1 = Annual Mean Temperature; BIO2 = Mean Diurnal Range (Mean of monthly (max temp - min temp)); BIO3 = Isothermality (BIO2/BIO7) (×100); BIO4 = Temperature Seasonality (standard deviation ×100); BIO5 = Max Temperature of Warmest Month; BIO6 = Min Temperature of Coldest Month; BIO7 = Temperature Annual Range (BIO5-BIO6); BIO8 = Mean Temperature of Wettest Quarter; BIO9 = Mean Temperature of Driest Quarter; BIO10 = Mean Temperature of Warmest Quarter; BIO11 = Mean Temperature of Coldest Quarter; BIO12 = Annual Precipitation; BIO13 = Precipitation of Wettest Month; BIO14 = Precipitation of Driest Month; BIO15 = Precipitation Seasonality (Coefficient of Variation); BIO16 = Precipitation of Wettest Quarter; BIO17 = Precipitation of Driest Quarter; BIO18 = Precipitation of Warmest Quarter; BIO19 = Precipitation of Coldest Quarter.
