# Supplementary material for: Geographic variation in the skull morphology of the lesser grison (Galictis cuja: Carnivora, Mustelidae) from two Brazilian ecoregions
Source: PeerJ. 2020 Nov 4;8:e9388. doi: 10.7717/peerj.9388 (PMC7648447; doi:10.7717/peerj.9388)
Supplement: Supplemental Information 4 — Significance (p < 0.05) is highlighted in bold. [file peerj-08-9388-s004.docx]

**Table S4. Results of spatial autocorrelation in the OLS residuals of *Galictis cuja* specimens in ventral (A = male, A’ = female), dorsal (B = male, B’ = female) and lateral (C = male, C’ = female) skull views.** Significance (*p* < 0.05) is highlighted in bold.

**A**

| Observed | Confidence interval (95%) | Expected value | *p* value | Mean distance |
| --- | --- | --- | --- | --- |
| -0.289026477 | 0.4251382 | -0.08333333 | 0.3429768 | 55.1275 |
| -0.051517492 | 0.4220466 | -0.08333333 | 0.8825370 | 113.1950 |
| 0.013681949 | 0.4354674 | -0.06666667 | 0.7176196 | 159.1075 |
| -0.098463252 | 0.4310396 | -0.06666667 | 0.8850396 | 218.5200 |
| 0.051341058 | 0.4350082 | -0.07692308 | 0.5633216 | 302.7833 |
| -0.085712925 | 0.4410471 | -0.06250000 | 0.9178379 | 399.5908 |
| -0.003408436 | 0.4159088 | -0.07692308 | 0.7290098 | 497.8008 |
| 0.014672734 | 0.4037060 | -0.07142857 | 0.6759300 | 706.9183 |
| 0.022495378 | 0.4206266 | -0.06250000 | 0.6920650 | 935.6950 |
| -0.071754839 | 0.4440808 | -0.07692308 | 0.9818014 | 1129.8917 |
| 0.042584543 | 0.4094468 | -0.08333333 | 0.5466669 | 1296.8437 |
| 0.015836857 | 0.3549584 | -0.09090909 | 0.5555749 | 1470.4471 |

**A’**

| Observed | Confidence interval (95%) | Expected value | *p* value | Mean distance |
| --- | --- | --- | --- | --- |
| 0.110917130 | 0.5627520 | -0.12500000 | 0.9608802 | 108462.5 |
| 0.231737714 | 0.5511365 | -0.10000000 | 0.2380978 | 197462.4 |
| -0.217704519 | 0.5883952 | -0.08333333 | 0.6544396 | 277124.9 |
| -0.022384226 | 0.5747196 | -0.11111111 | 0.7622020 | 375670.8 |
| -0.083517090 | 0.5904246 | -0.07692308 | 0.9825359 | 476524.9 |
| -0.219617151 | 0.5821485 | -0.08333333 | 0.6463451 | 586947.0 |
| -0.329270613 | 0.5864591 | -0.09090909 | 0.4256683 | 735677.0 |
| -0.201907394 | 0.5864591 | -0.09090909 | 0.7106625 | 900890.5 |
| 0.197158241 | 0.5934992 | -0.09090909 | 0.3414383 | 1047776.5 |
| 0.075007569 | 0.5849297 | -0.10000000 | 0.5575929 | 1181476.3 |
| 0.054873515 | 0.5793334 | -0.09090909 | 0.6218631 | 1389112.5 |
| -0.005011207 | 0.3875387 | -0.10000000 | 0.6309348 | 1833624.2 |

**B**

| Observed | Confidence interval (95%) | Expected value | *p* value | Mean distance |
| --- | --- | --- | --- | --- |
| -0.255472613 | 0.4241862 | -0.08333333 | 0.4263873 | 55.1275 |
| -0.057803238 | 0.4211201 | -0.08333333 | 0.9054151 | 113.1950 |
| 0.012656555 | 0.4345225 | -0.06666667 | 0.7204906 | 159.1075 |
| -0.048751429 | 0.4301316 | -0.06666667 | 0.9349368 | 218.5200 |
| -0.113688735 | 0.4341176 | -0.07692308 | 0.8681621 | 302.7833 |
| 0.068993993 | 0.4400748 | -0.06250000 | 0.5581131 | 399.5908 |
| 0.043134565 | 0.4150734 | -0.07692308 | 0.5707692 | 497.8008 |
| -0.057596576 | 0.4030070 | -0.07142857 | 0.9463659 | 706.9183 |
| -0.022655233 | 0.4198260 | -0.06250000 | 0.8524296 | 935.6950 |
| 0.085791175 | 0.4431144 | -0.07692308 | 0.4716952 | 1129.8917 |
| -0.005881619 | 0.4086257 | -0.08333333 | 0.7102635 | 1296.8437 |
| -0.054509202 | 0.3545639 | -0.09090909 | 0.8405300 | 1470.4471 |

**B’**

| Observed | Confidence interval (95%) | Expected value | *p* value | Mean distance |
| --- | --- | --- | --- | --- |
| -0.22485734 | 0.4890074 | -0.12500000 | 0.6889796 | 62.2850 |
| 0.01022335 | 0.5307509 | -0.11111111 | 0.6541000 | 171.2125 |
| -0.20647432 | 0.5315522 | -0.09090909 | 0.6700165 | 251.5975 |
| 0.08683416 | 0.5353178 | -0.09090909 | 0.5151853 | 351.6300 |
| 0.19083666 | 0.5538785 | -0.07142857 | 0.3533695 | 447.2450 |
| -0.46708686 | 0.5264527 | -0.10000000 | 0.1717269 | 550.7300 |
| 0.27884472 | 0.5278312 | -0.07692308 | 0.1864762 | 694.9500 |
| -0.16851409 | 0.5648422 | -0.08333333 | 0.7675533 | 866.0250 |
| 0.19977936 | 0.5512930 | -0.09090909 | 0.3013801 | 1025.8625 |
| -0.23398261 | 0.5660282 | -0.07692308 | 0.5865420 | 1167.4183 |
| 0.14724674 | 0.5156674 | -0.08333333 | 0.3808063 | 1384.8058 |
| -0.01471696 | 0.3546672 | -0.09090909 | 0.6737104 | 1832.7750 |

**C**

| Observed | Confidence interval (95%) | Expected value | *p* value | Mean distance |
| --- | --- | --- | --- | --- |
| -0.23705561 | 0.4062444 | -0.07692308 | 0.4397660 | 55.1400 |
| 0.02106940 | 0.4212978 | -0.08333333 | 0.6271714 | 112.8708 |
| -0.12539567 | 0.4165203 | -0.06250000 | 0.7672566 | 158.0958 |
| 0.03138590 | 0.4231635 | -0.06666667 | 0.6497152 | 210.0317 |
| -0.01038283 | 0.4113500 | -0.06666667 | 0.7885599 | 275.5517 |
| 0.05711767 | 0.4418328 | -0.05555556 | 0.6171976 | 361.5625 |
| -0.16228397 | 0.4032407 | -0.06666667 | 0.6421037 | 458.6525 |
| -0.03456496 | 0.3842266 | -0.05882353 | 0.9015158 | 652.6667 |
| 0.12524942 | 0.4017101 | -0.06250000 | 0.3596376 | 897.9267 |
| -0.07375305 | 0.4121994 | -0.08333333 | 0.9636657 | 1102.3725 |
| 0.15011809 | 0.3780313 | -0.07692308 | 0.2391345 | 1269.6775 |
| -0.12475221 | 0.3420510 | -0.09090909 | 0.8462340 | 1466.7450 |

**C’**

| Observed | Confidence interval (95%) | Expected value | *p* value | Mean distance |
| --- | --- | --- | --- | --- |
| -0.1257601385 | 0.4853163 | -0.12500000 | 0.9975506 | 62.2850 |
| -0.0311630046 | 0.5268930 | -0.11111111 | 0.7661607 | 171.2125 |
| -0.3847971799 | 0.5280234 | -0.09090909 | 0.2753169 | 251.5975 |
| 0.1505241270 | 0.5312488 | -0.09090909 | 0.3730641 | 351.6300 |
| 0.0714150219 | 0.5500678 | -0.07142857 | 0.6107663 | 447.2450 |
| -0.3595396631 | 0.5228986 | -0.10000000 | 0.3306318 | 550.7300 |
| 0.3141996552 | 0.5241289 | -0.07692308 | 0.1435718 | 694.9500 |
| -0.0823027549 | 0.5606412 | -0.08333333 | 0.9971253 | 866.0250 |
| 0.1776401977 | 0.5473019 | -0.09090909 | 0.3361853 | 1025.8625 |
| 0.0005170367 | 0.5618760 | -0.07692308 | 0.7870561 | 1167.4183 |
| -0.1014956366 | 0.5121653 | -0.08333333 | 0.9445876 | 1384.8058 |
| -0.0631562735 | 0.3560675 | -0.09090909 | 0.8785817 | 1832.7750 |
