# Supplementary figures and images for: Geographic variation in the skull morphology of the lesser grison (Galictis cuja: Carnivora, Mustelidae) from two Brazilian ecoregions
Source: PeerJ. 2020 Nov 4;8:e9388. doi: 10.7717/peerj.9388 (PMC7648447; doi:10.7717/peerj.9388)

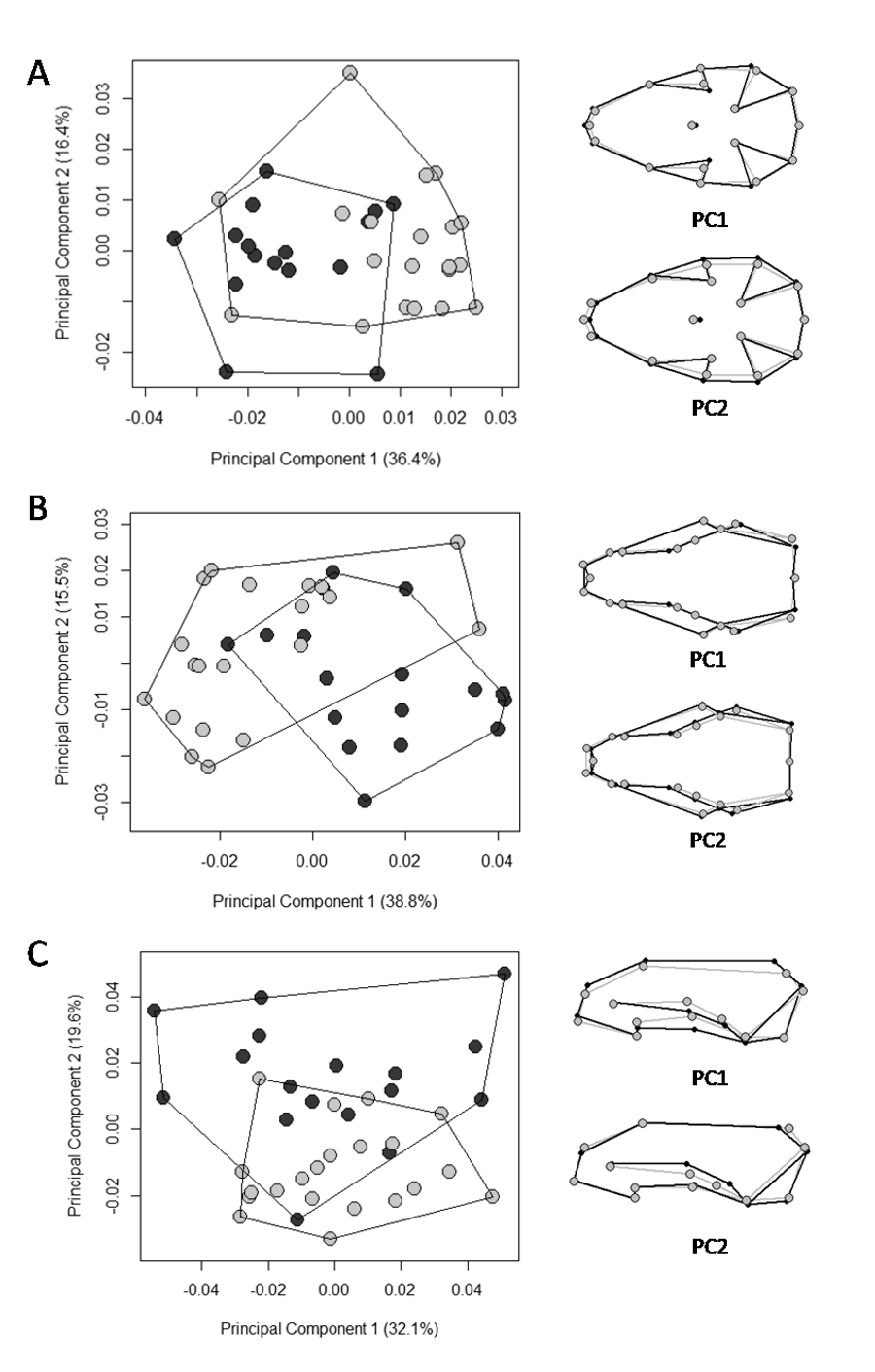

Supplement: Supplemental Information 5 — PC1 represents the skull shape variation in the first axis and PC2 represents skull shape variation in the second axis, in the ventral (A), dorsal (B) and lateral (C) views. Positive scores are represented by black lines and negative scores are represented by gray lines. [file peerj-08-9388-s005.png]
